# Methicillin-Resistant *Staphylococcus aureus*: Global Epidemiology and Molecular Epidemiological Trends

**DOI:** 10.3390/antibiotics15100971

**Published:** 2026-10-01

**Authors:** Hidemasa Nakaminami

**Affiliations:** Department of Clinical Microbiology, School of Pharmacy, Tokyo University of Pharmacy and Life Sciences, Tokyo 192-0392, Japan; nakami@toyaku.ac.jp; Tel.: +81-426-76-5619; Fax: +81-426-76-5647

**Keywords:** methicillin-resistant *Staphylococcus aureus*, healthcare-associated MRSA, community-associated MRSA, antimicrobial resistance

## Abstract

Since its emergence in the 1960s, methicillin-resistant *Staphylococcus aureus* (MRSA) has been recognized worldwide as a major cause of healthcare-associated infections. Traditionally, healthcare-associated MRSA (HA-MRSA) has been characterized as multidrug-resistant strains carrying staphylococcal cassette chromosome (SCC) *mec* types I–III, whereas community-associated MRSA (CA-MRSA) has been characterized as strains carrying SCC*mec* types IV or V that remain relatively susceptible to non-β-lactam antimicrobial agents. However, in recent years, the distinction between these two categories has become increasingly blurred owing to the global replacement of predominant clones, with community-associated clones entering healthcare settings and healthcare-associated clones spreading into the community. This review summarizes the epidemiology of MRSA in North America, South America, Europe, Africa, the Middle East, Asia, Oceania, and Japan and highlights the characteristics of the major circulating clones and their antimicrobial resistance profiles. Although the proportion of MRSA among *S. aureus* isolates has declined in many high-income countries owing to improvements in infection prevention and control, high isolation rates continue to be reported in Asia, the Middle East, and Africa. From a molecular epidemiological perspective, classical healthcare-associated clones, including ST239, ST5-II, and ST228-I, are declining, whereas SCC*mec* type IV clones, such as EMRSA-15 (ST22-IV), USA300 (ST8-IV), the Asian-Pacific clone (ST59-IV), and Queensland CA-MRSA (ST93-IV), are becoming predominant worldwide. In addition, although CA-MRSA has traditionally been considered susceptible to non-β-lactam antimicrobial agents, several clones have acquired multidrug resistance or novel resistance mechanisms. Moreover, with the widespread adoption of whole-genome sequencing (WGS), it has become possible to investigate the evolution of circulating clones, the acquisition of virulence factors and antimicrobial resistance genes, and even international transmission pathways at high resolution. Continued monitoring of regional molecular epidemiological characteristics, together with WGS-based international surveillance, will contribute to improved control of MRSA infections, optimization of infection prevention and control strategies, more appropriate empirical antimicrobial therapy, and the development of novel diagnostic and therapeutic approaches.

## 1. General Characteristics and Properties of *Staphylococcus aureus*

*Staphylococcus aureus* is a facultatively anaerobic, Gram-positive coccus and one of the best-characterized species of the genus *Staphylococcus*, typically forming grape-like clusters. It is coagulase-positive and catalase-positive and primarily colonizes the anterior nares and skin of humans; a substantial proportion of healthy individuals carry the organism in their nasal cavities and on their skin [1]. Despite being a common commensal organism, *S. aureus* is highly pathogenic and causes a wide spectrum of diseases ranging from opportunistic infections to life-threatening invasive infections, making it a major cause of both healthcare-associated and community-associated infections. Clinical manifestations range from skin and soft tissue infections (SSTIs), postoperative infections, and catheter-associated bloodstream infections (BSIs) to infective endocarditis, bone and joint infections, and pneumonia, and disease severity is influenced by the interaction between host factors (e.g., skin barrier disruption, immunosuppression, and the presence of indwelling medical devices) and bacterial virulence factors [2]. The pathogenicity of *S. aureus* is mediated by adhesins, including microbial surface components recognizing adhesive matrix molecules (MSCRAMMs), immune evasion factors, iron acquisition systems, and a wide variety of toxins [3,4,5,6]. Major toxins include pore-forming toxins such as α-hemolysin and Panton–Valentine leukocidin (PVL), exfoliative toxins, and superantigens, including staphylococcal enterotoxins and toxic shock syndrome toxin-1 (TSST-1), all of which contribute to tissue destruction, immune dysregulation, and the development of severe diseases, including necrotizing infections and toxic shock syndrome [7].

## 2. Methicillin-Resistant *Staphylococcus aureus* and the Staphylococcal Cassette Chromosome *mec*

Methicillin-resistant *S. aureus* (MRSA) is *S. aureus* that has acquired resistance to β-lactam antibiotics and first emerged in hospitals under selective antimicrobial pressure. MRSA emerged through the acquisition of the *mecA* gene, which encodes penicillin-binding protein 2a (PBP2a), a protein with low affinity for β-lactam antibiotics [8]. The *mecA* gene is located on a mobile genetic element known as the staphylococcal cassette chromosome *mec* (SCC*mec*). SCC*mec* comprises the *mec* gene complex and the cassette chromosome recombinase (*ccr*) gene complex, which mediates chromosomal integration and excision; the combination of these components determines the SCC*mec* type and subtype [9]. The SCC*mec* classification and nomenclature system has been updated according to the recommendations of the International Working Group on the Classification of Staphylococcal Cassette Chromosome Elements (IWG-SCC), and 15 SCC*mec* types (types I–XV) have been described to date [10]. A divergent *mecA* homolog, *mecC*, encodes an alternative penicillin-binding protein (PBP2c) and is usually found in SCC*mec* type XI. Although MRSA carrying *mecC* remains uncommon in humans, it is epidemiologically important due to its association with animal reservoirs and potential diagnostic challenges, as discussed below [11].

SCC*mec* type I was originally defined using the NCTC10442 strain, which was first isolated in the United Kingdom and serves as the prototype of type I [9]. SCC*mec* type II was originally defined using the Japanese MRSA strain N315, a representative healthcare-associated epidemic clone, and remains one of the most prevalent SCC*mec* types among HA-MRSA strains. SCC*mec* type III, represented by strain 85/2082, was established together with types I and II as one of the earliest SCC*mec* types identified in epidemic MRSA clones. SCC*mec* type IV is a relatively small cassette that became recognized in the early 2000s because of its close association with community-associated MRSA (CA-MRSA) and was originally identified in strain CA05 isolated in Japan [12]. SCC*mec* type IV is now recognized worldwide as the SCC*mec* type most frequently associated with CA-MRSA. SCC*mec* type V was originally identified in the Australian WIS strain and is characterized by the presence of the novel *ccrC* gene complex [13]. Like type IV, SCC*mec* type V is commonly associated with CA-MRSA.

## 3. Anti-MRSA Agents and Antimicrobial Resistance

The treatment of serious MRSA infections primarily relies on glycopeptides, lipoglycopeptides, oxazolidinones, lipopeptides, and anti-MRSA cephalosporins. vancomycin (VAN) is one of the most widely used agents for treating invasive MRSA infections. teicoplanin (TEC) is also used in several countries. vancomycin and teicoplanin inhibit cell-wall synthesis by binding to the D-Ala-D-Ala terminus of peptidoglycan precursors. While the majority of MRSA isolates remain susceptible to vancomycin, reduced susceptibility is clinically significant. Vancomycin-Intermediate *S. aureus* (VISA) and heterogeneous VISA (hVISA) predominantly result from cell-wall thickening and alterations in cell-wall regulatory pathways. In contrast, high-level Vancomycin-Resistant *S. aureus* (VRSA) mainly occurs from the acquisition of the *vanA* operon, which alters the glycopeptide target from D-Ala-D-Ala to D-Ala-D-Lac. VRSA remains exceptionally rare worldwide, whereas VISA and hVISA have been reported in multiple geographic regions [14,15].

Linezolid (LZD), an oxazolidinone antibiotic, inhibits protein synthesis by binding to the 50s ribosomal subunit. it is particularly useful for treating MRSA pneumonia and skin and soft tissue infections due to its excellent oral bioavailability and tissue penetration. tedizolid (TZD) is more potent than linezolid against MRSA in vitro and can be administered once daily. Resistance to oxazolidinones remains uncommon among MRSA isolates worldwide. The major mechanisms are mutations in domain V of the 23S rRNA (particularly G2576T), mutations in ribosomal proteins, and the acquisition of transferable resistance genes such as *cfr*, *optrA*, and *poxtA* [16]. The latter mechanisms are of particular epidemiological concern because they can be carried by mobile genetic elements and spread horizontally.

Daptomycin (DAP), a cyclic lipopeptide, has rapid bactericidal activity. it is an important alternative to VAN for treating MRSA bacteremia and other invasive infections; however, it is not suitable for treating pneumonia because it is inactivated by pulmonary surfactant. DAP resistance or non-susceptibility is rare worldwide, but it can develop during prolonged therapy, especially in cases of persistent MRSA bacteremia. Unlike resistance mediated by the acquisition of a single determinant, dap resistance generally develops through stepwise adaptive mutations that affect the bacterial cell membrane and cell wall. mutations in *mprF* are most frequently implicated, while alterations in *walKR*, *cls*, *rpoB*, and *rpoC* have also been associated with reduced susceptibility [17,18]. Importantly, reduced susceptibility to VAN and DAP may overlap because adaptive changes in the cell envelope can affect susceptibility to both agents.

Several newer agents have expanded the therapeutic options available against MRSA. Ceftaroline (CPT) and ceftobiprole (BPR), advanced-generation cephalosporins, have a high affinity for PBP2a and retain activity against MRSA despite conventional β-lactam resistance. CPT resistance is primarily associated with amino acid substitutions in the allosteric and transpeptidase domains of PBP2a, and its prevalence varies geographically. In the global atlas surveillance program, which involved bloodstream isolates collected between 2017 and 2020, 89.8% of MRSA isolates were susceptible to CPT. VAN; however, they remained active against all *S. aureus* isolates tested, and DAP and LZD also showed high activity [19]. Long-acting lipoglycopeptides, including dalbavancin and oritavancin, exhibit potent activity against MRSA and offer the advantage of infrequent dosing due to their long-acting nature. Tigecycline, delafloxacin, and trimethoprim-sulfamethoxazole are other agents that may be useful for selected MRSA infections, depending on the infection site, severity, and susceptibility profile.

## 4. Healthcare-Associated MRSA and Community-Associated MRSA

MRSA has traditionally been classified into healthcare-associated MRSA (HA-MRSA) and community-associated MRSA (CA-MRSA) according to the epidemiological characteristics of the host [20]. HA-MRSA is typically associated with healthcare exposure, including hospitalization, surgery, dialysis, residence in long-term care facilities, and the use of indwelling medical devices, and is a major cause of healthcare-associated infections [21]. From a molecular epidemiological perspective, HA-MRSA generally carries the larger SCC*mec* types I–III and exhibits multidrug resistance, including resistance to many non-β-lactam antimicrobial agents. In contrast, CA-MRSA typically causes skin and soft tissue infections (SSTIs) in otherwise healthy individuals without prior healthcare exposure, primarily carries SCC*mec* types IV or V, and remains susceptible to many non-β-lactam antimicrobial agents. Some CA-MRSA clones produce toxins such as Panton–Valentine leukocidin (PVL) and have been associated with severe SSTIs and necrotizing pneumonia [22]. However, in recent years, the distinction between community and healthcare settings has become increasingly blurred because of population aging and the expansion of home healthcare services and long-term care facilities, resulting in increasing detection of CA-MRSA among hospitalized patients [23]. Consequently, distinguishing HA-MRSA from CA-MRSA solely on the basis of epidemiological background has become increasingly difficult. Therefore, integrated evaluation of molecular epidemiological characteristics, including SCC*mec* type, sequence type (ST), and clonal complex (CC), is essential.

## 5. Livestock-Associated MRSA

Livestock-associated MRSA (LA-MRSA) is another epidemiologically important MRSA population primarily associated with food-producing animals, particularly pigs, cattle, and poultry. Humans who have frequent occupational contact with livestock, such as farmers, veterinarians, and slaughterhouse workers, are at an increased risk for LA-MRSA colonization. Zoonotic transmission can occasionally result in human infections [24]. The predominant LA-MRSA lineage varies by region: CC398 is widely distributed among livestock in Europe and North America, while ST9 is the predominant livestock-associated lineage in many Asian countries [24,25]. Genomic analyses suggest that the livestock-associated CC398 lineage originated from a human-associated, methicillin-susceptible *S. aureus* (MSSA) lineage. Although LA-MRSA is predominantly associated with exposure to livestock, these strains can be transmitted to humans and cause infections ranging from skin and soft tissue infections to invasive disease.

Another important population of MRSA associated with animals carries *mecC*, a divergent homolog of *mecA*. *mecC* encodes the alternative penicillin-binding protein PBP2c and is located within SCC*mec* type XI [11,26]. *mecC*-MRSA is most frequently associated with CC130, including ST130 and related lineages. It has been detected in humans, livestock, and wildlife, particularly in Europe. This supports the existence of animal reservoirs and zoonotic transmission [11,27]. Importantly, *mecC*-MRSA has diagnostic implications because conventional molecular assays that target only *mecA*, as well as some PBP2a-based tests, may fail to identify these isolates [28]. Phenotypic detection can also be challenging because oxacillin susceptibility can vary; cefoxitin-based susceptibility testing is generally more reliable for detecting *mecC*-mediated methicillin resistance [29]. Thus, the epidemiological boundaries among HA-MRSA, CA-MRSA, and LA-MRSA are not absolute. Therefore, integrated One Health surveillance that combines molecular epidemiological and genomic data from humans, animals, and their environments is important for monitoring the emergence and cross-species transmission of LA-MRSA.

## 6. Molecular Epidemiological Characteristics of MRSA

MRSA no longer fits neatly into the traditional epidemiological classifications of HA-MRSA and CA-MRSA because of clonal evolution, replacement of predominant clones, acquisition and loss of virulence and antimicrobial resistance determinants, and bidirectional transmission between healthcare settings and the community [30]. The increasing use of whole-genome sequencing (WGS) has revealed more about this epidemiological overlap. It has shown that MRSA lineages historically found in the community can spread in healthcare settings. It has also shown that lineages found in healthcare settings can spread outside of hospitals [31,32].

Compared to conventional typing methods, such as pulsed-field gel electrophoresis (PFGE) and multilocus sequence typing (MLST), which primarily classify isolates into broader genetic groups or lineages, WGS provides substantially higher resolution. WGS can distinguish closely related isolates within the same sequence type or clone [33]. This resolution allows for the reconstruction of transmission networks and the differentiation between local transmission and repeated introductions. It also enables the identification of the acquisition or loss of antimicrobial resistance and virulence determinants during clonal evolution [32,33,34]. Consequently, WGS has shifted the understanding of MRSA epidemiology from a relatively static classification based on healthcare- or community-associated clones to a more dynamic framework that considers the evolution, transmission, and movement of individual lineages across epidemiological settings [31,32].

Several complementary analytical approaches are used to interpret WGS data. Core-genome MLST (cgMLST) expands upon the seven-locus MLST method by incorporating hundreds or thousands of conserved loci. This approach provides substantially greater discriminatory power while maintaining an allele-based methodology that enables standardized comparisons among isolates [35,36]. In contrast, single-nucleotide polymorphism (SNP)-based phylogenomic analysis provides a high level of detail for investigating closely related isolates and is particularly useful for reconstructing outbreaks and transmission pathways [37]. Comparative studies have shown that cgMLST and SNP-based approaches generally provide highly concordant assessments of genetic relatedness. However, the inferred distances and thresholds for defining transmission clusters may differ between methods [38].

WGS enables the simultaneous characterization of the resistome and virulome. It can also identify plasmids, prophages, and other mobile genetic elements involved in the dissemination of antimicrobial resistance and virulence determinants [35]. Genomic antimicrobial resistance prediction can complement phenotypic susceptibility testing by identifying known resistance genes and chromosomal mutations. In *S. aureus*, WGS-based resistance prediction has demonstrated high concordance with phenotypic susceptibility testing across multiple antimicrobial agents [39]. However, genotype-phenotype discordance and novel or incompletely characterized resistance mechanisms remain important limitations. Additionally, differences in sequencing platforms, genome assembly, bioinformatic pipelines, reference genomes, and thresholds used to define genomic relatedness can affect comparisons among studies [38,40]. Therefore, standardization of analytical and reporting frameworks is essential for integrating WGS into regional and international MRSA surveillance [35,40].

The major circulating MRSA clones, as well as their antimicrobial resistance patterns and toxin gene profiles, differ considerably among countries and regions [41]. Although particular MRSA lineages are often linked to specific antimicrobial resistance profiles, the lineage itself should not be considered the direct cause of resistance. Rather, resistance phenotypes reflect the combined effects of lineage-specific genetic backgrounds; acquired resistance determinants carried by plasmids and other mobile genetic elements; chromosomal mutations; local antimicrobial selection pressures; and temporal evolution. Thus, the resistance patterns associated with specific clones in this review are presented as epidemiological associations observed in the cited studies, not as intrinsic properties of the lineages. Studies based on WGS have demonstrated that genetically distinct sublineages may circulate in different geographical areas, even when they belong to the same conventional sequence type. This emphasizes the importance of integrating genomic and epidemiological information when investigating regional and international MRSA transmission [34]. Importantly, substantial regional variation in the molecular epidemiological characteristics of MRSA can also be observed within the same country [23]. This review summarizes the global epidemiology and molecular epidemiological characteristics of MRSA and describes the major circulating clones and their antimicrobial resistance characteristics in North America, South America, Europe, Africa, the Middle East, Asia, Oceania, and Japan.

## 7. Literature Search and Study Selection

This article is a narrative review and was not designed as a systematic review, scoping review, or meta-analysis. Relevant literature on the epidemiology and molecular epidemiology of MRSA was identified primarily through PubMed searches using combinations of terms, including “methicillin-resistant *Staphylococcus aureus*,” “MRSA,” “epidemiology,” “prevalence,” “molecular epidemiology,” “clone,” “sequence type,” “SCC*mec*,” “whole-genome sequencing,” “healthcare-associated MRSA,” and “community-associated MRSA,” together with geographical terms for individual countries and regions. Reference lists of relevant articles were also examined to identify additional studies. The literature search was last updated in July 2026.

Peer-reviewed studies reporting MRSA proportions among *S. aureus* isolates, MRSA carriage prevalence, incidence, molecular epidemiological characteristics, major clones, or antimicrobial resistance were considered. Studies focusing exclusively on single case reports and studies lacking sufficient epidemiological or molecular information were generally excluded. Additionally, only English-language papers were included. Recent studies, particularly those published since 2010, were prioritized for regional epidemiological comparisons; however, earlier studies were included when they provided important longitudinal data or documented the emergence or replacement of major MRSA clones. When multiple studies were available from the same country or region, priority was given to nationwide or multicenter surveillance studies, studies with larger sample sizes, and studies providing detailed molecular characterization using MLST, SCC*mec* typing, *spa* typing, or WGS.

Due to substantial heterogeneity in study populations, clinical settings, specimen types, denominators, sampling periods, and definitions of HA-MRSA and CA-MRSA, epidemiological measures were not quantitatively pooled. Instead, MRSA proportions, carriage prevalence, and incidence measures are presented according to the definitions and denominators used in the original studies, together with their geographical setting and study period. When overlapping datasets were identified, the study providing the most comprehensive or relevant epidemiological or molecular information was included. Thus, the studies summarized in this review are representative rather than exhaustive. The review aims to provide a global narrative overview of temporal and geographical trends in MRSA epidemiology and molecular characteristics.

## 8. MRSA Isolated in North America

In North America, the proportion of MRSA among clinical *S. aureus* isolates has generally declined in both healthcare and community settings. However, substantial variation remains according to healthcare facility, patient population, specimen type, and geographic region (Table 1 and Table 2). In the United States, MRSA accounted for 30% to 40% of *S. aureus* bloodstream isolates in recent hospital-based studies [31,42,43], while national surveillance in Canada documented a decline in the proportion of MRSA among clinical *S. aureus* isolates, from 26.1% in 2007 to 16.9% in 2016 [44]. Similar downward trends have been observed among community-associated isolates in the United States [45,46]. These declines have coincided with strengthened infection prevention and surveillance. Notably, the implementation of a nationwide MRSA prevention initiative in the U.S. Department of Veterans Affairs healthcare system was associated with significant reductions in hospital-onset MRSA infections and MRSA acquisition [47,48].

The molecular epidemiology of MRSA in North America has undergone substantial clonal replacement, particularly the expansion of CC8/USA300 (ST8-IV) into healthcare settings (Table 1 and Table 2) [31,32,42,43,44,46,47,48,49,50,51,52,53,54,55,56,57,58,59,60,61,62]. In the United States, CC8/USA300 and CC5, including the New York/Japan clone (ST5-II), are prevalent among hospitalized patients. USA300, initially identified as the primary community-associated lineage, has also become a significant cause of healthcare-associated and invasive infections [54,55,60,61,62]. Studies of the WGS have demonstrated close genetic overlap between community- and healthcare-associated USA300 isolates. This indicates that conventional epidemiological categories do not necessarily correspond to genetically distinct populations [31,32]. In Canada, the historically dominant USA100/800 lineage remains prevalent, though USA300 has increased in both community and healthcare settings [44,49,50,63]. Thus, the expansion of USA300 illustrates the increasingly blurred distinction between HA-MRSA and CA-MRSA in North America.

The antimicrobial resistance patterns of North American MRSA have evolved alongside changes in clonal composition. The expansion of USA300 and the decline of classical multidrug-resistant healthcare-associated lineages have been accompanied by reductions in multidrug resistance in some settings. However, substantial lineage- and region-specific differences remain [44,46,54]. Notably, USA300, which was traditionally susceptible to many non-β-lactam antimicrobial agents, has acquired additional resistance determinants, including *tetK* and *ermC* [64]. *ermC*-carrying plasmids have been identified within USA300 transmission networks linking correctional facilities and surrounding communities [59]. Despite these emerging resistance traits, resistance to key anti-MRSA agents, including vancomycin, linezolid, and daptomycin, remains uncommon. This underscores the importance of continued genomic and antimicrobial resistance surveillance.

## 9. MRSA Isolated in South America

The proportion of MRSA among clinical *S. aureus* isolates in South America varies considerably by country, healthcare setting, specimen type, and study period (Table 3 and Table 4). Among hospitalized patients, studies of bloodstream infections have generally reported MRSA proportions ranging from approximately 30% to over 60%. In a prospective, multicenter study conducted at 24 hospitals in nine Latin American countries, MRSA accounted for 45% of blood-derived *S. aureus* isolates [65,66,67]. Community-associated MRSA also shows substantial geographic variation. In Argentina, for example, MRSA was isolated from 70% of patients with community-associated SSTIs in a prospective multicenter study. However, considerably lower proportions have been reported in some other South American settings [68,69,70,71].

The molecular epidemiology of MRSA in South America has shifted from the predominance of classical healthcare-associated CC5 lineages to greater clonal diversity involving SCC*mec* type IV lineages (Table 3 and Table 4) [65,66,67,72,73,74]. In Chile and Argentina, the previously dominant ST5-I Chilean/Córdoba clone has declined, while lineages such as ST105-II, ST30-IV, ST5-IV, and ST8-IV have increased [66,72]. Genomic analyses have identified CC30-IVc, CC5-IV, and CC8-IVc (USA300-LV) as major epidemic lineages circulating across the continent [74]. ST30 remains important in Argentina, while USA300-LV has become particularly prevalent in northern South America, including Colombia, Venezuela, and Ecuador. It has also been detected in Chile [68,79,80]. These changes illustrate the progressive replacement of classical healthcare-associated clones by more diverse lineages, as well as the increasing overlap between community- and healthcare-associated MRSA populations.

Antimicrobial resistance patterns vary substantially across South America and should be interpreted in the context of circulating lineages and acquired resistance determinants. In Brazilian hospitals, multidrug resistance was more frequently observed among CC5-II than CC5-IV isolates. However, these differences should not be interpreted as intrinsic properties of the clonal background or SCC*mec* type alone [73]. High levels of resistance to macrolides, clindamycin, fluoroquinolones, and aminoglycosides have been reported among bloodstream MRSA isolates in some settings, including Peru, whereas susceptibility to linezolid and TMP-SMX has remained preserved [67]. CA-MRSA generally retains greater susceptibility to non-β-lactam agents, particularly TMP-SMX and tetracyclines. However, resistance patterns vary considerably among countries and circulating lineages [68,79,81]. Continued surveillance is therefore important as community-associated lineages, including USA300-LV, become increasingly established in community and healthcare settings.

## 10. MRSA Isolated in Europe

Over the past two decades, MRSA proportions have declined in many European countries. However, substantial geographic variation persists, with lower rates in northwestern Europe and higher rates in southern and southeastern Europe (Table 5 and Table 6) [82,83,84,85]. In the Netherlands, MRSA accounts for approximately 1% of bloodstream *S. aureus* isolates. This low level has been associated with the long-standing “search-and-destroy” strategy and restrictive antimicrobial use [82,86,87,88]. CA-MRSA is less prevalent overall, but it also varies considerably across Europe. In a prospective multicenter study of community-associated SSTIs in seven European countries, MRSA accounted for 16.6% of *S. aureus* isolates, with substantial variation among the participating countries [89,90,91,92].

The molecular epidemiology of MRSA in Europe has undergone substantial clonal replacement. Classical healthcare-associated lineages, such as ST239-III, ST228-I, and ST247-I, have declined, while CC22-IV (EMRSA-15), CC5 lineages, and ST8-IV have become increasingly prominent (Table 5 and Table 6) [84,93,94,95,96,97,101,102]. This transition is particularly evident in Germany and Italy, where ST228-I was largely replaced by CC22-IV and related lineages. In contrast, greater persistence of ST239-III has been observed in Greece [93,94,102]. Community-associated MRSA has also become more diverse. Although ST80-IV was historically the dominant CA-MRSA lineage in Europe, ST8/USA300-related, ST22, ST5, and other lineages now circulate across the continent [89,90,98,99,100]. The increasing detection of traditionally community-associated lineages in healthcare settings, including ST80-IV in Greece, illustrates the diminishing epidemiological distinction between HA-MRSA and CA-MRSA [102].

The antimicrobial resistance patterns of European MRSA have changed alongside clonal replacement. However, resistance should be interpreted in relation to specific resistance determinants and local antimicrobial selection rather than lineage identity alone. In several settings, the decline of classical multidrug-resistant, healthcare-associated clones has coincided with an increased susceptibility to certain non-β-lactam agents. However, resistance to fluoroquinolones, macrolides, and aminoglycosides remains common [84,93,94,95,96,97,101,102,103,104]. Resistance to key anti-MRSA agents is uncommon; however, ceftobiprole resistance has been reported among multiple genetic lineages in Italy [103]. CA-MRSA is generally more susceptible to non-β-lactam agents than classical HA-MRSA, but resistance to macrolides, tetracyclines, aminoglycosides, and fluoroquinolones has been increasingly documented [98,100]. These trends underscore the importance of continued surveillance as increasingly diverse MRSA lineages circulate across community and healthcare settings.

## 11. MRSA Isolated in Africa

The epidemiology of MRSA in Africa is highly heterogeneous, varying substantially according to country, healthcare setting, study population, and specimen type (Table 7 and Table 8). Among hospitalized patients, high MRSA proportions have been reported in several countries. For instance, MRSA accounted for 46% of *S. aureus* bacteremia cases in a large South African surveillance study [105]. Other hospital-based studies have likewise documented substantial but highly variable MRSA proportions across the continent [106,107]. In community settings, MRSA carriage appears relatively uncommon in the general population. Substantially higher proportions, however, have been reported among patients with SSTIs and chronic wound infections [108,109,110,111]. However, direct comparisons among countries remain difficult because available studies differ considerably in sampling strategy, patient population, and surveillance intensity [112,113].

The molecular epidemiology of MRSA in Africa is characterized by significant regional variation and an apparent shift from traditional healthcare-associated lineages to a wider variety of region-specific clones (Table 7 and Table 8). ST239/ST241-III has been reported in multiple African countries historically, but more recent studies indicate an increase in clonal diversity. This includes ST1, ST5, ST22, ST88, ST152, and other lineages [105,108,112,114,115,116,117,118]. Distinct regional patterns have emerged, including ST612 in South Africa and ST80-IV in North Africa [105,107]. Community-associated MRSA is similarly diverse. ST88-IV is widely distributed, while ST152 and other PVL-positive lineages have been reported in several sub-Saharan African countries [109,111,117,119,120]. Overall, African CA-MRSA populations consist of several distinct, geographically isolated lineages, none of which predominate across the continent.

Antimicrobial resistance patterns among African MRSA isolates are heterogeneous. Multidrug resistance is common among healthcare-associated isolates. Resistance determinants affecting aminoglycosides, fluoroquinolones, TMP-SMX, and tetracyclines have been reported in multiple lineages. However, susceptibility to glycopeptides and linezolid is generally preserved [105,106,117]. Community-associated isolates also exhibit variable resistance to tetracyclines, TMP-SMX, macrolides, and other non-β-lactam agents [109,110]. However, uneven surveillance capacity across the continent limits interpretation of these resistance patterns and clonal distributions. Much of the available evidence comes from small, single-center, or geographically restricted studies. Limited access to molecular typing and WGS may result in underrepresentation of some regions [112,113]. Therefore, expansion of standardized, multicenter, and genomic surveillance will be essential for defining contemporary MRSA transmission and resistance patterns in Africa.

## 12. MRSA Isolated in the Middle East

The proportion of MRSA among clinical *S. aureus* isolates varies substantially by country, healthcare setting, and study population in the Middle East (Table 9 and Table 10) [121,122,123,124,125]. Recent studies continue to document a significant MRSA presence in several countries, though temporal trends are inconsistent. Notably, nationwide surveillance in the United Arab Emirates revealed an increase in the proportion of MRSA, rising from 21.9% in 2010 to 33.5% in 2021 [123]. Community-associated MRSA is also widely distributed across the region. However, nasal carriage among healthy individuals is generally low to moderate and varies considerably according to the population studied [121,126,127].

The molecular epidemiology of MRSA in the Middle East has shifted from the historical predominance of ST239-III to increasing clonal diversity and dissemination of SCC*mec* type IV lineages, particularly ST22-IV, ST80-IV, and ST30-IV, in healthcare settings (Table 9 and Table 10) [124,128,129,130,131,135]. ST80-IV is also an important community-associated lineage, particularly in Lebanon, Jordan, and Saudi Arabia, where it is frequently associated with PVL-positive SSTIs [132,136,137]. Other region-specific lineages have also become established, including the ST22-IVa Gaza strain [126]. These findings collectively indicate an increasing overlap between traditionally healthcare- and community-associated MRSA populations throughout the Middle East.

The antimicrobial resistance patterns of Middle Eastern MRSA isolates vary greatly and reflect differences in circulating lineages, acquired resistance determinants, and local antimicrobial selection pressures. Resistance to macrolides, lincosamides, fluoroquinolones, aminoglycosides, tetracyclines, and fusidic acid has been reported among healthcare-associated isolates, with substantial variation among countries and studies [125,135]. Community-associated isolates generally exhibit greater susceptibility to non-β-lactam agents; however, ST80-IV isolates are often associated with fusidic acid and aminoglycoside resistance [127,132,137]. The ongoing growth of SCC*mec* type IV lineages in community and healthcare settings underscores the need for comprehensive molecular and antimicrobial resistance surveillance throughout the region.

## 13. MRSA Isolated in Asia

MRSA remains a leading cause of healthcare-associated *S. aureus* infections in many Asian countries, though its prevalence varies by country, healthcare setting, patient population, and specimen type (Table 11 and Table 12) [138,139,140]. For example, in China, MRSA accounted for 44.2% of *S. aureus* bloodstream isolates collected at tertiary care hospitals in Shanghai, while substantial MRSA burdens have also been documented in Republic of Korea, Hong Kong, and India [138,139,140]. MRSA carriage prevalence varies widely among community populations across the region, with marked differences reported in studies from China, Nepal, and Taiwan [141,142,143,144].

The molecular epidemiology of MRSA in Asia is highly heterogeneous, characterized by region-specific clonal replacement rather than the expansion of a single dominant lineage (Table 11 and Table 12). CC5 lineages, particularly ST5 and ST764, remain important in China, with SCCmec type II predominating among CC5 MRSA in some settings [138,139,145,146,147,148,149,154]. In Singapore, the historically dominant ST239-III lineage has progressively been replaced by ST22-IV (EMRSA-15). Meanwhile, ST59-IV/V is being detected more frequently in community and healthcare settings in China and Taiwan [148,149,155]. ST59 is particularly important among CA-MRSA in East Asia, while ST72 is prominent in Republic of Korea, and ST22 and ST772 are important in India [36,150,151,152,153,156,157,158,159,160,161]. The coexistence and movement of these lineages between community and healthcare settings increasingly challenge the conventional epidemiological distinction between HA-MRSA and CA-MRSA.

The antimicrobial resistance patterns of Asian MRSA isolates vary substantially by region and lineage. Classical healthcare-associated lineages, such as ST5 and ST239, are often multidrug resistant. In contrast, several SCC*mec* type IV lineages, including ST59, ST72, and ST22, generally exhibit greater susceptibility to non-β-lactam agents; however, resistance continues to evolve [138,145,149,154,161]. Importantly, multidrug resistance has also emerged among community-associated lineages, particularly ST772 in India [156,162]. Genomic analyses of ST772 have identified acquired resistance determinants, including *aacA-aphD*, *dfrG*, and blaZ, as well as mutations in *gyrA* and *grlA* that are associated with fluoroquinolone resistance [162]. These findings underscore the fact that resistance phenotypes reflect the acquisition and evolution of specific resistance mechanisms rather than clonal identity alone, and they highlight the need for continued genomic and antimicrobial resistance surveillance across Asia.

Due to the extensive nationwide and longitudinal molecular epidemiological data available in Japan, it is discussed separately in the following section as a case study illustrating the dynamic clonal replacement of MRSA in Asia.

## 14. MRSA Isolated in Oceania

Epidemiological data on MRSA in Oceania is predominantly derived from Australia. There, the proportion of MRSA among *S. aureus* bloodstream isolates has gradually declined over the past decade, reaching 14.9% in 2024 (Table 13 and Table 14) [163,164]. Conversely, community-associated lineages have become more prevalent, accounting for 75% of MRSA bloodstream isolates in 2017 and 89.6% in 2024 [163,164]. Notably, marked geographical and population-specific differences persist. In Central Australia, for instance, MRSA accounted for 57% of *S. aureus* isolates from patients requiring surgical treatment for furuncles and carbuncles, primarily among Indigenous Australians [165]. Molecular epidemiological data from other parts of Oceania remain comparatively limited [166,167].

The molecular epidemiology of MRSA in Australia has undergone substantial clonal replacement. Among healthcare-associated lineages, ST22-IV (EMRSA-15) has progressively replaced the historically dominant ST239-III (Aus-2/3 EMRSA) [163,164,168,169]. Even more striking is that community-associated lineages now account for the vast majority of MRSA bloodstream isolates. PVL-positive ST93-IV (Queensland CA-MRSA) remains the predominant CA-MRSA lineage in Australia [163,164,165]. Other SCC*mec* types IV and V also contribute to the genetically diverse CA-MRSA population (Table 14) [163,164]. In New Zealand, a separate clonal transition has occurred, with PVL-negative ST5-IV replacing the previously predominant ST30-IV [171]. These trends demonstrate that community-associated lineages have become a major cause of invasive MRSA infection, further blurring the distinction between CA-MRSA and HA-MRSA.

The antimicrobial resistance patterns of Australian MRSA have changed in parallel with these epidemiological shifts. Resistance to macrolides and fluoroquinolones is relatively common among bloodstream isolates, whereas resistance to TMP-SMX is uncommon [163,164]. ST93-IV generally remains susceptible to many non-β-lactam agents; however, ST45-V exhibits multidrug resistance involving resistance to fluoroquinolones, macrolides, lincosamides, aminoglycosides, and tetracyclines [164,165]. Resistance to vancomycin and linezolid is still rare. However, in 2023, daptomycin-resistant MRSA isolates were detected, followed in 2024 by a daptomycin-resistant ST5-V isolate carrying an MprF S337L substitution [164]. These findings underscore the importance of ongoing genomic and antimicrobial resistance surveillance, particularly as community-associated lineages increasingly contribute to invasive MRSA infections.

## 15. MRSA in Japan: A Case Study of Clonal Replacement

Japan is a well-characterized example of longitudinal MRSA clonal replacement, as evidenced by long-term, nationwide molecular epidemiological surveillance (Table 15 and Table 16). Despite substantial changes in the circulating MRSA population, MRSA remains prevalent among *S. aureus* bloodstream isolates. A nationwide study conducted from 2019 to 2020 found that MRSA accounted for 46.4% of *S. aureus* bloodstream isolates. Of those, 76.5% carried SCC*mec* type IV [172]. Community-associated lineages have also become increasingly prevalent. Among outpatient MRSA isolates collected from 244 healthcare facilities in 31 prefectures, the proportion carrying SCC*mec* type IV increased from 44.0% to 75.8% between 2010 and 2018 [173,174,175,176]. These findings suggest that SCC*mec* type IV MRSA is widely established in healthcare and community settings in Japan.

The molecular epidemiology of MRSA in Japan has undergone significant changes, with the prevalence of the classical ST5-II lineage declining and the SCC*mec* type IV lineages, particularly ST1-IV, ST8-IV, and ST2725-IV, becoming predominant among bloodstream isolates (Table 15) [172,177,178,179]. There are geographic differences, with ST1-IV predominating in eastern Japan and ST8-IV in western Japan. Meanwhile, ST764-II persists as an important SCC*mec* type II lineage [172,179]. Similar diversification has occurred in community settings (Table 16). Among PVL-positive MRSA causing SSTIs, the ΨUSA300 lineage has recently become predominant. Meanwhile, ST22-PT, a lineage producing both PVL and TSST-1, has been identified at multiple institutions throughout Japan [176,180,181]. These findings demonstrate the ongoing clonal replacement and diversification of MRSA in healthcare and community settings in Japan.

Antimicrobial resistance patterns in Japanese MRSA have changed alongside clonal replacement. The decline of classic healthcare-associated lineages has coincided with reduced resistance to certain non-β-lactam agents. However, resistance to fluoroquinolones and macrolides remains prevalent among hospital-associated isolates [23,174]. In contrast, major PVL-positive community-associated lineages, including USA300 and ST22-PT, generally exhibit greater susceptibility to several non-β-lactam agents, particularly clindamycin, minocycline, and TMP-SMX. However, macrolide and fluoroquinolone resistance has also been observed [23]. These lineage-associated resistance patterns should be interpreted as epidemiological associations reflecting specific resistance determinants and clonal evolution, rather than the lineages’ intrinsic properties. Continued molecular and antimicrobial resistance surveillance is warranted as community-associated lineages become increasingly established in outpatient and hospital settings.

## 16. Drivers of Global MRSA Clonal Replacement

The global replacement of MRSA clones is unlikely to be explained by a single genetic factor. Rather, epidemic success appears to result from the interaction of bacterial fitness, antimicrobial resistance, virulence, host adaptation, mobile genetic elements (MGEs), and transmission opportunities. Methicillin resistance itself can impose a biological cost. The experimental introduction of SCC*mec* into *S. aureus* has demonstrated an inverse relationship between methicillin resistance levels and bacterial growth. This suggests a fitness cost associated with resistance [182]. Additionally, compensatory evolution may partially mitigate the costs of antimicrobial resistance on fitness. These fitness-resistance trade-offs may contribute to the decline of some highly multidrug-resistant classical HA-MRSA lineages and the expansion of fitter lineages. ST239 is a representative example. Genomic and competition analyses demonstrated that ST239 isolates had lower competitive fitness than ST8 and ST30 isolates. This suggests that fitness costs contributed to the global decline of this once-dominant lineage [183].

However, achieving epidemic success requires more than just high intrinsic fitness. Antimicrobial selection can strongly favor specific genetic backgrounds, and the balance between resistance and fitness varies depending on the ecological setting. A well-characterized example is EMRSA-15 (ST22-IV). Genomic reconstruction revealed that the acquisition of fluoroquinolone resistance preceded the rapid expansion of a major EMRSA-15 clade in the United Kingdom. This suggests that antimicrobial selection contributed to its epidemic success [184]. In Singapore, ST22 subsequently spread rapidly through hospitals and displaced the previously dominant multidrug-resistant ST239 lineage [185]. Thus, clonal replacement may occur when a lineage achieves a favorable balance between antimicrobial resistance, biological fitness, and efficient transmission within a particular healthcare environment.

Virulence, colonization capacity, and MGEs provide an additional layer of selective advantage. SCC*mec*, plasmids, transposons, prophages, and other genomic islands can introduce antimicrobial resistance, virulence, or host-adaptation determinants into successful genetic backgrounds. USA300 is a representative example of this process. In addition to SCC*mec*, USA300 commonly carries ACME. Experimental studies demonstrated that the ACME-encoded arginine deiminase system promotes growth under acidic conditions resembling the human skin environment. The ACME-encoded SpeG protein enhances resistance to host polyamines, suggesting a role for ACME in skin adaptation and persistence [186]. However, the contribution of ACME alone does not fully explain USA300’s epidemic success, suggesting that its successful clonal expansion likely reflects the combined effects of multiple genetic and epidemiological factors.

Host adaptation and ecological reservoirs also influence clonal expansion. LA-MRSA CC398 exemplifies how host switching, antimicrobial selection, and horizontal gene transfer can reshape a lineage. Phylogenomic reconstruction suggests that livestock-associated CC398 originated from a human-associated MSSA population. Its transition to livestock was accompanied by the acquisition of methicillin and tetracycline resistance, as well as the loss of prophage-associated human immune evasion genes [187]. More recent, large-scale genomic analyses have further demonstrated that antimicrobial resistance determinants can remain stably associated with livestock populations. However, transmission back to humans can be accompanied by the rapid acquisition of human-adaptive elements, particularly φSa3 prophages carrying immune-evasion genes [188]. These findings demonstrate that animal reservoirs can act as sources of zoonotic transmission and as ecological environments in which resistance and host-adaptation traits are selected and exchanged.

Together, these observations support a model in which the replacement of MRSA clones is driven by the dynamic selection of the optimal combination of resistance, fitness, virulence, host adaptation, and transmissibility within a particular ecological niche. In healthcare settings, antibiotic use and infection-control practices shape selection pressures, while host behavior and contact networks influence transmission in the community. Antimicrobial exposure and host adaptation, in turn, shape MRSA populations in livestock reservoirs. Consequently, the success of clones such as USA300, ST22, ST59, and CC398, as well as the decline of previously dominant clones such as ST5 and ST239, should not be attributed to a single “epidemic” determinant. Rather, clonal success represents an evolving phenotype generated by interactions between the bacterial core genome, horizontally acquired elements, antimicrobial pressure, the host population, and the transmission environment. This framework may also explain why the same clone can expand in one geographical or epidemiological setting but remain uncommon in another.

## 17. Knowledge Gaps and Future Research Priorities

Despite substantial advances in MRSA molecular epidemiology, several major knowledge gaps remain. Surveillance data are disproportionately derived from high-income countries and regions with well-established laboratory and genomic infrastructure, while many low- and middle-income countries are underrepresented. Available studies also differ substantially in their sampling strategies, patient populations, specimen types, epidemiological definitions, and molecular typing methods, which limits direct comparisons across regions. Community-based surveillance is less comprehensive than hospital-based surveillance in many settings, and animal and environmental reservoirs are incompletely characterized. These limitations are particularly relevant to LA-MRSA and *mecC*-MRSA, highlighting the need for standardized One Health surveillance that integrates human, animal, and environmental populations.

There are also important technological and methodological gaps. Although WGS provides substantially greater resolution than conventional molecular typing, access to sequencing and bioinformatic capacity remains uneven globally. Differences in sequencing platforms, analytical pipelines, reference genomes, nomenclature, and thresholds for defining genomic relatedness can hinder interoperability among surveillance systems. Genomic prediction of antimicrobial resistance can complement phenotypic antimicrobial susceptibility testing; however, genotype-phenotype discordance and novel or incompletely characterized resistance mechanisms remain important limitations. Therefore, greater integration of genomic data with phenotypic antimicrobial susceptibility data is required. Particular attention should be directed toward resistance determinants carried by plasmids and other mobile genetic elements because horizontal transfer may facilitate their dissemination across otherwise distinct MRSA lineages.

Several fundamental biological questions remain unanswered. The mechanisms that enable particular MRSA lineages to expand successfully, cross ecological boundaries between community and healthcare settings, and replace previously dominant clones are not fully understood. Future studies should move beyond descriptive molecular epidemiology and investigate the genetic and phenotypic determinants of epidemic success. These include colonization capacity, environmental persistence, transmissibility, virulence, antimicrobial resistance, and adaptation to specific host populations or healthcare environments. In particular, the relative contributions of resistance-associated fitness costs, compensatory evolution, antimicrobial selection, and mobile genetic elements to clonal emergence and replacement require further investigation. Additionally, the increasing movement of MRSA among community, healthcare, livestock, and other settings may generate epidemiologically complex or hybrid populations that cannot be adequately described using the conventional HA-, CA-, and LA-MRSA categories.

Longitudinal, population-level genomic studies combining historical and contemporary isolates will be essential for reconstructing the evolutionary processes underlying clonal emergence and replacement. Genomic data should be integrated with phenotypic, clinical, epidemiological, and, when applicable, transcriptomic information to identify genetic changes that confer a selective advantage and contribute to successful dissemination. These integrated datasets are important for distinguishing local transmission from repeated introductions and improving transmission attribution. Prospective studies are also necessary to determine if genomic surveillance and rapid identification of emerging clones lead to measurable improvements in outbreak control, antimicrobial use, and patient outcomes. Therefore, establishing interoperable international surveillance frameworks that combine standardized epidemiological data, phenotypic antimicrobial susceptibility testing, and genomic information is an important priority for future MRSA research.

## 18. Limitations

In addition to the broader knowledge gaps mentioned above, the limitations of this narrative review must be acknowledged. First, the quantity, recency, and methodological quality of the available epidemiological data varied substantially by geographical region. Notably, molecular epidemiological studies from several African countries and other resource-limited settings were fewer, older, smaller, and/or geographically restricted compared to those from North America and Europe. Consequently, the apparent predominance of certain MRSA clones or high MRSA proportions in these regions may reflect a lack of recent surveillance data rather than the current epidemiological situation. Second, differences in study populations, specimen sources, definitions of HA-MRSA and CA-MRSA, sampling periods, antimicrobial susceptibility testing, and molecular typing methods limited direct comparisons among studies and regions. Finally, many earlier studies relied on conventional typing methods, such as MLST, *spa* typing, or SCC*mec* typing, rather than WGS. Consequently, genetically distinct sublineages may have been grouped within the same conventional clone. Therefore, the regional MRSA proportions and clonal distributions summarized in this review should be interpreted in the context of these differences in data availability and methodology.

## 19. Conclusions

Since its emergence in the 1960s, MRSA has been one of the most significant antimicrobial-resistant pathogens in healthcare settings worldwide. However, as this review has shown, the proportion of MRSA and its molecular epidemiological characteristics vary greatly by region and continue to evolve over time. While the proportion of MRSA among *S. aureus* isolates has generally declined in many European countries and the United States, it remains high in many parts of Asia, the Middle East, and Africa. These findings demonstrate substantial regional heterogeneity and underscore the importance of surveillance and control strategies tailored to local epidemiological conditions.

Classical HA-MRSA lineages, such as ST239, ST5-II, and ST228-I, which once predominated in many regions, have declined and are being replaced by SCC*mec* type IV lineages. These include EMRSA-15, USA300, the Asian-Pacific clone, the Taiwan clone, and the Queensland CA-MRSA clone. Furthermore, SCC*mec* type IV lineages that were historically considered community-associated are now often found in BSIs and other healthcare-associated infections. Thus, the traditional epidemiological distinction between HA-MRSA and CA-MRSA is becoming increasingly blurred. Surveillance based solely on presumed infection origin may no longer adequately capture the changing MRSA population structure.

There are also significant regional and lineage-specific differences in antimicrobial resistance. Classical HA-MRSA lineages are often associated with multidrug-resistant phenotypes, while many recently emerging SCC*mec* type IV lineages demonstrate greater susceptibility to non-β-lactam antimicrobial agents. However, these patterns are not intrinsic properties of individual clones and can change through the acquisition or loss of MGEs, chromosomal mutations, antimicrobial selection, and temporal evolution.

Global MRSA surveillance should increasingly incorporate standardized epidemiological and genomic approaches. Currently, differences in study populations, specimen sources, epidemiological definitions, antimicrobial susceptibility testing methods, and molecular typing methods limit direct comparisons among countries and regions. Standardized reporting of essential metadata, including patient setting, infection type, geographical location, antimicrobial susceptibility, and molecular characteristics, would improve the comparability of surveillance data. WGS should also be progressively integrated with epidemiological and clinical information to facilitate the detection of emerging clones, reconstruction of transmission networks, identification of resistance and virulence determinants, and recognition of international dissemination. As discussed in Section 17, strengthening genomic surveillance in underrepresented regions and improving the interoperability of surveillance systems are important priorities.

Similarly, infection prevention and control strategies should evolve in response to the changing epidemiology of MRSA. As community-associated lineages increasingly circulate within healthcare facilities, traditional approaches based on a strict distinction between HA-MRSA and CA-MRSA may become less informative. Therefore, infection control programs should incorporate local information on circulating clones and transmission patterns alongside conventional measures, such as hand hygiene, epidemiologically appropriate screening, contact precautions, environmental cleaning, and antimicrobial stewardship. Rapid molecular or genomic investigation of suspected outbreaks may facilitate the recognition of transmission events that cannot be identified through conventional epidemiological investigation alone. Integrating molecular surveillance with antimicrobial stewardship is also important because the expansion of particular clones may be influenced by antimicrobial selection pressure, transmission fitness, and virulence.

In conclusion, the global MRSA population is not static. Rather, it is continuously reshaped by clonal replacement, antimicrobial selection, the acquisition and loss of resistance and virulence determinants, host adaptation, and transmission across community, healthcare, and animal-associated settings. Therefore, future MRSA surveillance should move beyond monitoring MRSA proportions alone and adopt an integrated framework that combines standardized epidemiological data, phenotypic antimicrobial susceptibility, and genomic information. Along with efforts to address the knowledge gaps outlined in Section 17, this approach is essential for the early detection of emerging MRSA threats and for developing more effective strategies for infection prevention, antimicrobial stewardship, and public health control.

## Figures and Tables

**Table 1:** Molecular epidemiological characteristics of HA-MRSA in North America.

Timeframe	No. of Isolates	Analysis Method	Disease	Country	Region	Major Clones	Antimicrobial Resistance	Reference
1995–2008	37,169	PFGE, SCC*mec* typing	MRSA infection/colonization	Canada	National	USA100/800, USA300	ND	[49]
2007–2016	1963	*spa* typing	BSI, respiratory tract infection, wound/IV-site infection, UTI	Canada	National	USA100/800, USA300	CLR, CLI, FQs, TMP-SMX, TGC	[44]
2008–2012	1753	*spa* typing	BSI	Canada	National	USA100/800, USA300	ND	[50]
2009–2012	535	PFGE, *spa* typing	Blood culture isolates	Canada	Quebec	USA100/800, USA300	ND	[51]
2009–2013	60	WGS	BSI	USA	Illinois	USA300, USA100	FQs, ERY, CLI	[31]
2010–2018	323	WGS	BSI	USA	New Hampshire	ST5, ST8	ERY, CLI, FQs, GEN, TET, TMP-SMX	[43]
2010–2022	852	WGS	BSI	USA	New Hampshire	ST8, ST5, ST105	ERY, CLI, FQs, GEN, TET, TMP-SMX	[52]
2011–2013	2211	PFGE, *spa* typing	BSI	Canada	Alberta	USA300, USA100/800	ND	[53]
2011–2014	516	*spa* typing, SCC*mec* typing	Blood culture and nasal carriage	USA	Multistate	USA300, USA100	CLI, ERY, LVX, MXF	[54]
2015	152	WGS	BSI	USA	Minnesota	CC5, CC8	ERY, CLI, FQs, GEN, TET, TMP-SMX	[42]
2018–2019	104	WGS	BSI	USA	Pennsylvania	CC8, CC5	ND	[55]

PFGE, pulsed-field gel electrophoresis; WGS, whole-genome sequencing; BSI, bloodstream infection; UTI, urinary tract infection; IV, intravenous; CC, clonal complex; ST, sequence type; CLI, clindamycin; CLR, clarithromycin; ERY, erythromycin; FQs, fluoroquinolones; GEN, gentamicin; LVX, levofloxacin; MXF, moxifloxacin; TET, tetracycline; TGC, tigecycline; TMP-SMX, trimethoprim-sulfamethoxazole; ND, not described.

**Table 2:** Molecular epidemiological characteristics of CA-MRSA in North America.

Timeframe	No. of Isolates	Analysis Method	Disease	Country	Region	Major Clones	Antimicrobial Resistance	Reference
2005–2008	823	PFGE, SCC*mec* typing	Health care-associated, community-onset infections; Community-associated infections	USA	Multistate	USA300	ERY, LVX; emerging CLI, MUP and TET resistance	[56]
2007–2016	1963	*spa* typing	Community-associated infections, including SSTI	Canada	National	USA300, USA400	FQs, ERY and CLI	[44]
2007–2017	275	WGS	SSTI and BSI	USA	Illinois	USA300, USA100	ND	[57]
2010–2012	597	*spa* typing, SCC*mec* typing	Closed skin abscess	USA	Multistate	USA300	ND	[58]
2010–2019	382,149	Phenotypic antimicrobial susceptibility testing	Community-onset infection in outpatients	USA	National	ND	TET 3.6 to 12.8%; TMP-SMX 2.6 to 9.2%; CLI 24.2 to 30.6%; macrolides 73.5 to 60.2%	[46]
2011–2014	1020	WGS	Primarily wound infection	USA	Illinois	USA300	ND	[32]
2015–2018	308	WGS	Wound infection and asymptomatic carriage in jail detainees	USA	Illinois	USA300	ND	[59]

PFGE, pulsed-field gel electrophoresis; WGS, whole-genome sequencing; SSTI, skin and soft tissue infection; BSI, bloodstream infection; ERY, erythromycin; LVX, levofloxacin; CLI, clindamycin; MUP, mupirocin; TET, tetracycline; FQs, fluoroquinolones; TMP-SMX, trimethoprim-sulfamethoxazole; ND, not described.

**Table 3:** Molecular epidemiological characteristics of HA-MRSA in South America.

Timeframe	No. of Isolates	Analysis Method	Disease	Country	Region	Major Clones	Antimicrobial Resistance	Reference
2000–2016	469	WGS	Respiratory tract infection, BSI/other sterile-site infection, SSTI	Chile	Santiago	ST5, ST105, ST72	ND	[72]
2009–2016	326	PFGE, SCC*mec* typing, MLST, *spa* typing	BSI	Argentina	Buenos Aires	ST5-I decreased, ST30-IV, ST5-IV and ST8-IV increased	Reduced MDR	[66]
2011–2014	1185	PFGE, SCC*mec* typing, MLST, WGS	BSI	Argentina, Brazil, Chile, Colombia, Ecuador, Guatemala, Mexico, Peru, Venezuela	Multinational	Chilean/Cordobes, New York/Japan, USA300-LV	FQs, ERY, and GEN resistance varied by clone	[65]
2014–2017	600	SCC*mec* typing, WGS	BSI, non-BSI infection, nasal carriage	Brazil	Rio de Janeiro	CC5-II, CC5-IV, CC30-IV, CC1-IV, ST105-II	MDR	[73]
2017–2019	166	SCC*mec* typing	BSI	Peru	National, 10 regions	Chilean/Cordobes	ERY, CLI, CIP, GEN	[67]
2019	443	WGS	BSI	Argentina, Bolivia, Brazil, Paraguay, Uruguay	Multinational	CC30-IVc, CC5-IV, USA300-LV	MLS_B_	[74]

PFGE, pulsed-field gel electrophoresis; WGS, whole-genome sequencing; MLST, multilocus sequence typing; BSI, bloodstream infection; SSTI, skin and soft tissue infection; ST, sequence type; CC, clonal complex; MDR, multidrug resistance; FQs, fluoroquinolones; ERY, erythromycin; GEN, gentamicin; CLI, clindamycin; CIP, ciprofloxacin; MLS_B_, macrolide-lincosamide-streptogramin B; ND, not described.

**Table 4:** Molecular epidemiological characteristics of CA-MRSA in South America.

Timeframe	No. of Isolates	Analysis Method	Disease	Country	Region	Major Clones	Antimicrobial Resistance	Reference
2006–2008	1570	PFGE, SCC*mec* typing, MLST	Community- and healthcare-associated infections	Colombia, Ecuador, Peru, Venezuela	Multination	USA300-LV	TET	[75]
2008	28	PFGE, SCC*mec* typing, MLST, *spa* typing	Nasal and oropharyngeal carriage in healthy children	Argentina	Buenos Aires	ST5-IV, ST239-III, ST5-I	ERY, CLI, GEN	[76]
2009–2013	39	PFGE, SCC*mec* typing, MLST, *spa* typing	Invasive infections in children	Paraguay	Asunción and Central Department	CC30-IV, CC5-IV, CC8-IV	CHL, ERY, CLI, GEN	[77]
2010–2011	218	PFGE, SCC*mec* typing, MLST, *spa* typing	SSTI in adults and adolescents	Argentina	Buenos Aires	ST30-IV, ST5-IV	Limited non-β-lactam resistance	[68]
2014	190	PFGE, SCC*mec* typing, MLST	Nasal and oral carriage in Indigenous populations	Brazil	São Paulo and Acre	ST5	ND	[78]
2007–2023	54	WGS	Community-associated infections	Chile	Nationwide	USA300-LV, USA300, ST30, ST5	Limited non-β-lactam resistance	[79]

PFGE, pulsed-field gel electrophoresis; WGS, whole-genome sequencing; MLST, multilocus sequence typing; SSTI, skin and soft tissue infection; ST, sequence type; CC, clonal complex; TET, tetracycline; ERY, erythromycin; CLI, clindamycin; GEN, gentamicin; CHL, chloramphenicol; ND, not described.

**Table 5:** Molecular epidemiological characteristics of HA-MRSA in Europe.

Timeframe	No. of Isolates	Analysis Method	Disease	Country	Region	Major Clones	Antimicrobial Resistance	Reference
1990–2019	784	PFGE, WGS (selected)	BSI	Spain	Barcelona	CC5-IV, CC8-IV, EMRSA-15	FQs, ERY, TOB	[84]
2000–2010	778	DNA microarray	Clinical MRSA isolates from hospitalized patients	Germany	Single center	ST228-I, EMRSA-15	ND	[93]
2006–2010	575	PFGE, SCC*mec* typing	Hospital-acquired MRSA infections	Italy	Milan	ST228-I, EMRSA-15	FQs, CLI, ERY	[94]
2013–2018	190	SCC*mec* typing, MLST, *spa* typing	Hospital-acquired MRSA infections	Spain	Valencia	ST125, CC5, CC8, CC22	MDR	[95]
2016	66	WGS	Hospital-acquired pneumonia	Italy	National	EMRSA-15, ST228-I, CC8	ERY, FQs	[96]
2020	412	SCC*mec* typing, *spa* typing	Clinical MRSA isolates	Slovakia	National	CC5-II, EMRSA-15, CC8-IV, CC45-IV, CC5-IV, CC5-I	ERY, CLI, FQs	[97]

PFGE, pulsed-field gel electrophoresis; WGS, whole-genome sequencing; MLST, multilocus sequence typing; BSI, bloodstream infection; ST, sequence type; CC, clonal complex; FQs, fluoroquinolones; ERY, erythromycin; TOB, tobramycin; CLI, clindamycin; MDR, multidrug-resistance; ND, not described.

**Table 6:** Molecular epidemiological characteristics of CA-MRSA in Europe.

Timeframe	No. of Isolates	Analysis Method	Disease	Country	Region	Major Clones	Antimicrobial Resistance	Reference
2000–2010	568	PFGE, SCC*mec* typing, MLST, *spa* typing	Community-associated MRSA isolates	Europe (16 countries)	Multicenter	USA300, European CA-MRSA, Taiwan clone	FUS, TET, ERY	[98]
2004–2012	8326	PFGE, SCC*mec* typing, MLST, *spa* typing	Community-associated MRSA infections	Spain	National	ST8-IVc, USA300	Mostly β-lactams only	[90]
2015	205	MLST, *spa* typing, DNA microarray	Community-acquired SSTI	Europe (7 countries)	Multicenter	Diverse lineages; no predominant clone	ND	[89]
2011–2012	11	*spa* typing	Nasal carriage in primary care patients	The Netherlands/Germany	Primary care	LA-MRSA (t011, t034); HA-MRSA spa types	ND	[99]
2018–2022	98	WGS	Pediatric CA-MRSA infections	Spain	Multicenter	ST8, ST8-IVc	ERY, TOB, TET	[100]

PFGE, pulsed-field gel electrophoresis; WGS, whole-genome sequencing; MLST, multilocus sequence typing; SSTI, skin and soft tissue infection; ST, sequence type; FUS, fusidic acid; TET, tetracycline; ERY, erythromycin; TOB, tobramycin; ND, not described.

**Table 7:** Molecular epidemiological characteristics of HA-MRSA in Africa.

Timeframe	No. of Isolates	Analysis Method	Disease	Country	Region	Major Clones	Antimicrobial Resistance	Reference
2006–2011	221	SCC*mec* typing, MLST	Hospital-associated infections	Algeria	Algiers	European CA-MRSA (ST80-IV), ST239-III	KAN, TET, FUS	[114]
2010–2012	2709	SCC*mec* typing, MLST, *spa* typing	BSI	South Africa	Multicenter	ST612, ST5, ST36, ST239	FQs, CLI, ERY, GEN, TET, TOB	[105]
2010–2012	308	SCC*mec* typing, MLST, *spa* typing	Mainly SSTI and invasive infections	Ghana	Multicenter	CC152, CC15, CC121, CC8, CC5, CC1	FQs, CLI, ERY, GEN, TET, FOS	[115]
2010–2017	2019	SCC*mec* typing, *spa* typing	BSI	South Africa	National	*spa* t037, *spa* t1257, *spa* t012, *spa* t045	FQs, ERY, GEN, TET, TOB, TMP-SMX	[116]
2010–2023	96	WGS	SSTI, wound infection, BSI and other clinical infections	Kenya	Nairobi	ST239/241 to ST152, ST4803	FQs, ERY, GEN, TET, TOB, TMP-SMX	[117]
2011–2012	64	SCC*mec* typing, *spa* typing	SSI	Uganda	Kampala	*spa* t645, *spa* t4353, *spa* t064, *spa* t355	FQs, CLI, ERY, TET	[118]

WGS, whole-genome sequencing; MLST, multilocus sequence typing; BSI, bloodstream infection; SSTI, skin and soft tissue infection; SSI, surgical site infection; ST, sequence type; CC, clonal complex; KAN, kanamycin; TET, tetracycline; FUS, fusidic acid; FQs, fluoroquinolones; CLI, clindamycin; ERY, erythromycin; GEN, gentamicin; TOB, tobramycin; TMP-SMX, trimethoprim-sulfamethoxazole.

**Table 8:** Molecular epidemiological characteristics of CA-MRSA in Africa.

Timeframe	No. of Isolates	Analysis Method	Disease	Country	Region	Major Clones	Antimicrobial Resistance	Reference
2006–2007	346	SCC*mec* typing, MLST, *spa* typing	Community-associated conjunctivitis, otitis and SSTI	Nigeria	Ibadan	ST88, ST241, ST250	TMP-SMX, TET, FQs, ERY	[111]
2012–2013	17	PFGE, SCC*mec* typing, *spa* typing	Nasal carriage in community outpatients and healthy volunteers	Morocco	Tangier	ST22, ST398	TET	[119]
2016	28	WGS	Chronic wound infection in outpatients	Ghana	Asante Akim North District	ST88	TET	[120]

PFGE, pulsed-field gel electrophoresis; WGS, whole-genome sequencing; MLST, multilocus sequence typing; SSTI, skin and soft tissue infection; ST, sequence type; TMP-SMX, trimethoprim-sulfamethoxazole; TET, tetracycline; FQs, fluoroquinolones; ERY, erythromycin.

**Table 9:** Molecular epidemiological characteristics of HA-MRSA in the Middle East.

Timeframe	No. of Isolates	Analysis Method	Disease	Country	Region	Major Clones	Antimicrobial Resistance	Reference
2003, 2008	78	PFGE, SCC*mec* typing, MLST	Hospital-associated clinical infections	United Arab Emirates	Abu Dhabi	ST239-III, ST22-IV, ST5-II, ST80-IV, ST5-IV	ND	[128]
2009–2015	117	DNA microarray	Nosocomial infections	Saudi Arabia	Riyadh	CC80, CC6, CC5, CC22, ST239-III	ND	[129]
2010–2011	107	DNA microarray	Clinical infections and nasal carriage	Saudi Arabia	Riyadh	ST239-III, CC22-IV, CC30-IV, CC80-IV	ND	[130]
2011	132	PFGE, SCC*mec* typing, MLST, *spa* typing	SSTI, respiratory, BSI and other clinical infections	Lebanon	Beirut	ST80-IV	CIP, CLI, ERY, TET	[124]
2016	1327	SCC*mec* typing, *spa* typing, DNA microarray	Hospital-associated infections	Kuwait	Multicenter (13 hospitals)	CC361-V, EMRSA-15, CC30-VI, CC1-V, CC8-V	FUS, KAN, ERY/CLI, TMP, CIP, TET	[131]

PFGE, pulsed-field gel electrophoresis; MLST, multilocus sequence typing; ST, sequence type; CC, clonal complex; CIP, ciprofloxacin; CLI, clindamycin; ERY, erythromycin; TET, tetracycline; FUS, fusidic acid; KAN, kanamycin; TMP, trimethoprim; ND, not described.

**Table 10:** Molecular epidemiological characteristics of CA-MRSA in the Middle East.

Timeframe	No. of Isolates	Analysis Method	Disease	Country	Region	Major Clones	Antimicrobial Resistance	Reference
2000–2011	94	PFGE, SCC*mec* typing, MLST, *spa* typing	Mainly SSTI, respiratory infection, and bacteremia	Lebanon and Jordan	Multicenter	ST80-IV	KAN, FUS, TET, ERY	[132]
2009	215	PFGE, SCC*mec* typing, MLST, *spa* typing	Nasal carriage in healthy children and parents	Palestine	Gaza Strip	ST22-IVa (Gaza strain)	ERY, CLI	[126]
2013–2014	25	SCC*mec* typing, MLST	Nasal carriage in healthy children	Iran	Isfahan	ST859-IV	TET	[133]
2017–2019	625	DNA microarray	Clinical MRSA infections	United Arab Emirates	Multicenter (Dubai, Abu Dhabi, Umm Al Quwain)	CC30, CC5, CC22, CC6, CC1, USA300, Bengal Bay	FUS, ERY/CLI, GEN	[134]

PFGE, pulsed-field gel electrophoresis; MLST, multilocus sequence typing; SSTI, skin and soft tissue infection; ST, sequence type; CC, clonal complex; KAN, kanamycin; FUS, fusidic acid; TET, tetracycline; ERY, erythromycin; CLI, clindamycin; GEN, gentamicin.

**Table 11:** Molecular epidemiological characteristics of HA-MRSA in Asia.

Timeframe	No. of Isolates	Analysis Method	Disease	Country	Region	Major Clones	Antimicrobial Resistance	Reference
2006–2010	494	SCC*mec* typing, MLST	Clinical infections, including BSI	Singapore	Multicenter	EMRSA-15 (ST22-IV), ST239-III, ST45-IV	CIP, ERY, CLI, GEN	[145]
2008–2018	1782	MLST, *spa* typing	BSI	Republic of Korea	Seoul	ST5, ST72, ST8	higher non-β-lactam resistance	[146]
2012–2014	80	PFGE, SCC*mec* typing, MLST, *spa* typing	Respiratory, wound and other clinical infections	China	Xiangyang	ST239-t030/t632, ST59-t437	ERY, CLI, TET, GEN, FQs	[147]
2013	666	MLST	MRSA colonization	Singapore	National	EMRSA-15, ST45, ST239	MUP	[148]
2013–2018	120	SCC*mec* typing, MLST, *spa* typing	BSI	China	Shanghai	ST5-II, ST764-II	MDR	[138]
2014–2020	565	WGS	Blood, wound/pus and respiratory specimens	China	Multicenter	ST59-t437-IV, ST239-t030-III, ST5-t2460-II, ST764-II	ERY, CLI, CIP, TET, GEN	[149]
2016–2021	74	SCC*mec* typing, MLST	Pediatric BSI	Republic of Korea	Seoul	ST72, ST5, ST1	MDR	[139]

PFGE, pulsed-field gel electrophoresis; WGS, whole-genome sequencing; MLST, multilocus sequence typing; BSI, bloodstream infection; ST, sequence type; CIP, ciprofloxacin; ERY, erythromycin; CLI, clindamycin; GEN, gentamicin; TET, tetracycline; FQs, fluoroquinolones; MUP, mupirocin; MDR, multidrug resistance.

**Table 12:** Molecular epidemiological characteristics of CA-MRSA in Asia.

Timeframe	No. of Isolates	Analysis Method	Disease	Country	Region	Major Clones	Antimicrobial Resistance	Reference
2006–2009	412	PFGE, SCC*mec* typing, MLST, *spa* typing	Mainly SSTI, wound infection, respiratory infection	India	Mumbai	EMRSA-15, Bengal Bay, ST239-III	CIP, GEN, ERY, CLI, TET	[150]
2007–2010, 2013–2014	637	MLST, *spa* typing	Nasal carriage in newly admitted patients	Republic of Korea	Seoul	ST72, ST5, ST239	ND	[151]
2011–2020	182	PFGE, SCC*mec* typing, MLST	Pediatric MRSA infections, mainly SSTI	Taiwan	Northern Taiwan	ST59-Vt, ST59-IV, ST45-IV, ST8-IV	ERY, CLI; low TMP-SMX resistance	[152]
2013–2015	292	WGS	MRSA infections, including SSTI	China	Zhejiang	ST5, ST59	FQs, ERY, CLI, TET	[36]
2015–2018	91	SCC*mec* typing, MLST, *spa* typing	Pediatric SSTI	China	Shanghai	ST59-t437, ST188, ST398-IV	ERY, CLI	[153]

PFGE, pulsed-field gel electrophoresis; WGS, whole-genome sequencing; MLST, multilocus sequence typing; SSTI, skin and soft tissue infection; ST, sequence type; CIP, ciprofloxacin; GEN, gentamicin; ERY, erythromycin; CLI, clindamycin; TET, tetracycline; TMP-SMX, trimethoprim-sulfamethoxazole; FQs, fluoroquinolones; ND, not described.

**Table 13:** Molecular epidemiological characteristics of HA-MRSA in Oceania.

Timeframe	No. of Isolates	Analysis Method	Disease	Country	Region	Major Clones	Antimicrobial Resistance	Reference
2015	2401	WGS	BSI	Australia	National	EMRSA-15 (ST22-IV), Aus-2/3 EMRSA (ST239-III)	ERY, CIP	[168]
2018	2673	WGS	BSI	Australia	National	EMRSA-15 (ST22-IV), Aus-2/3 EMRSA (ST239-III)	ERY, CIP	[163]
2021	2928	WGS	BSI	Australia	National	EMRSA-15 (ST22-IV), Aus-2/3 EMRSA (ST239-III)	ERY, CIP	[169]
2024	3358	WGS	BSI	Australia	National	EMRSA-15 (ST22-IV), Aus-2/3 EMRSA (ST239-III)	ERY, CIP	[164]

WGS, whole-genome sequencing; BSI, bloodstream infection; ERY, erythromycin; CIP, ciprofloxacin.

**Table 14:** Molecular epidemiological characteristics of CA-MRSA in Oceania.

Timeframe	No. of Isolates	Analysis Method	Disease	Country	Region	Major Clones	Antimicrobial Resistance	Reference
2005–2011	3323	Phage typing SCC*mec* typing, MLST, *spa* typing	Clinical MRSA infections	New Zealand	National	ST30-IV, ST5-IV, ST22-IV, ST1-IV, ST8-IV (USA300), ST93-IV, ST239-III	ND	[170]
2014	50	High-resolution melting analysis	Carbuncles and furuncles requiring surgical intervention	Australia	Central Australia	ST93-IV (Queensland CA-MRSA)	CLI	[165]
2017	478	WGS	BSI	Australia	National	ST93-IV, ST45-VT, ST5-IV, ST1-IV, ST78-IV	CIP, ERY, CLI, GEN, TET	[167]
2024	503	WGS	BSI	Australia	National	ST93-IV, ST5-IV, ST45-V, ST8-IV (USA300), ST30-IV, ST1-IV, ST6-IV, ST97-IV, ST22-IV	CIP, ERY, CLI, GEN, TET	[164]

WGS, whole-genome sequencing; MLST, multilocus sequence typing; BSI, bloodstream infection; CLI, clindamycin; CIP, ciprofloxacin; ERY, erythromycin; GEN, gentamicin; TET, tetracycline; ND, not described.

**Table 15:** Molecular epidemiological characteristics of HA-MRSA in Japan.

Timeframe	No. of Isolates	Analysis Method	Disease	Region	Major Clones	Antimicrobial Resistance	Reference
2010–2014	2589	PFGE, SCC*mec* typing, MLST	Clinical MRSA isolates	Tokyo (Multicenter)	CC1-IV (USA400/J), CC8-IV (CA-MRSA/J), CC5-II	LVX resistant	[177]
2010–2021	245	SCC*mec* typing, MLST	BSI	Fukuoka	CC5-II to CC1-IV	LVX, ERY, GEN, CLI, MIN	[178]
2019	270	WGS	BSI	National	ST8-IV, ST1-IV, ST2725-IV, ST764-II, ST5-II	Higher CLI and MIN susceptibility in SCCmec IV clones	[179]
2019–2020	580	WGS	BSI	National	ST1-IV, ST8-IV, ST2725-IV, ST764-II, ST5-II	GEN, ERY, CLI, MIN, LVX	[172]

PFGE, pulsed-field gel electrophoresis; WGS, whole-genome sequencing; MLST, multilocus sequence typing; BSI, bloodstream infection; CC, clonal complex; ST, sequence type; LVX, levofloxacin; CLI, clindamycin; MIN, minocycline; GEN, gentamicin; ERY, erythromycin.

**Table 16:** Molecular epidemiological characteristics of CA-MRSA in Japan.

Timeframe	No. of Isolates	Analysis Method	Disease	Region	Major Clones	Antimicrobial Resistance	Reference
2010 and 2012	673	PFGE, SCC*mec* typing, MLST	Outpatient SSTI	National	ST1-IV, ST5-IV, ST22-IV, ST30-IV, ST59-V, ST8-IV (USA300)	ND	[173]
2010–2018	1770	SCC*mec* typing, WGS	Outpatient SSTI	National	USA300 (ST8-IVa), ST22-PT, ST59	ND	[174]
2013–2017	1455	PFGE, SCC*mec* typing, MLST, *spa* typing	Outpatient SSTI	National	USA300	MIN, FUS susceptible	[175]
2018–2021	980	PFGE, SCC*mec* typing, MLST, *spa* typing	Outpatient SSTI	National	ΨUSA300, USA300, ST22-PT	ND	[176]

PFGE, pulsed-field gel electrophoresis; WGS, whole-genome sequencing; MLST, multilocus sequence typing; SSTI, skin and soft tissue infection; ST, sequence type; MIN, minocycline; FUS, fusidic acid; ND, not described.

## Data Availability

No new data were created or analyzed in this study. Data sharing is not applicable to this article.
